# Journalism – De-constructing the jigsaw

**DOI:** 10.4102/sajr.v25i1.2356

**Published:** 2021-12-09

**Authors:** Maya Patel

**Affiliations:** 1 Department of Radiology, School of Medicine, Faculty of Health Sciences, University of the Witwatersrand, Johannesburg, South Africa; 2 National Radiology Services Inc, Johannesburg, South Africa

Academic journalism is akin to building a puzzle in many respects. Every piece has a specific position and role to play and development occurs as the pieces interlock succinctly and mould into something recognisable.

Being openly accessible, published manuscripts are permanently under the scrutiny of the scholarly community microscope and therefore sound methodology, ethics, integrity and accuracy are vital components of literary journals. These pieces connect through four major cornerstones, namely: the authors, reviewers, editors and publishers. The interplay between these key role players is crucial in creating a solid framework on which to expand. With the foundation in place, growth can commence; but like a picturesque puzzle, the challenge lies in finding the correct place for all the elements. Occasionally, pieces do not fit compactly and this can be likened to oversights and differing opinions. Whilst these are not always welcome, they do indicate a positive interest in the published material and promote healthy constructive criticism.

Where do journal metrics fit? Research journals are continually measured, compared and ranked. These numerical ratings provide a locus for the journal which can change depending on the journal’s performance. The more commonly known metric is the journal impact factor, but others include the CiteScore, the SCImago Journal Rank (SJR), the Eigen factor and the Source Normalised Impact per paper (SNIP). The number of readers, downloads, citations and even popularity on social media, all affect these metrics and ultimately the journal’s prestige.

Closely linked to journal metrics is indexing, which involves a formal application for inclusion on a bibliographic or literature database, often linked to an affiliated institution. Selecting the appropriate database for one’s journal is cardinal and inclusion depends on whether all the desired criteria are met. The process of indexing is similar to the peer review process that manuscripts undergo, except that the entire journal undergoes a technical review by an external advisory committee over a certain period, usually 2 years. Ultimately, indexing is all about journal exposure.

Similar to matching puzzle pieces, author metrics are aligned with journal metrics and databases. Authors are graded according to their publication numbers, citations and first author publications, amongst other criteria. Most databases will provide authors with a profile calculation, such as the h-index, which allows them to gauge themselves against colleagues. An alternative is to consider author esteem and expertise. A broad range of influential editorial board members and collaborators are helpful in this regard. These pieces are like bridges between the focal point of the puzzle and the background. Additionally, like a visually stimulating puzzle image, journal content should spark interest and relevance, capturing the reader’s attention.

South Africa is a niche geographic location in Africa, and there is a wealth of information from our local disease burden that is unique to our country and worthy of dissemination. The *South African Journal of Radiology* puzzle is intricate and diverse and made up of several elements. Although expanding the journal is an onerous task, all puzzles can be solved and the pieces are finding their places with time.

Maya Patel

